# Prevenção e tratamento da instabilidade na artroplastia total do quadril

**DOI:** 10.1055/s-0045-1810123

**Published:** 2025-12-10

**Authors:** Bruno Alves Rudelli, Fabio Seiji Mazzi Yamaguchi, Marco Rudelli, Lucas Torres Oliveira, Helder de Souza Miyahara, Henrique Melo de Campos Gurgel

**Affiliations:** 1 Instituto de Ortopedia e Traumatologia, Hospital das Clínicas da Faculdade de Medicina da Universidade de São Paulo (IOT-HCFMUSP), São Paulo, SP, Brasil; 2 Departamento de Ortopedia e Traumatologia, Santa Casa de Misericórdia de São Paulo, São Paulo, SP, Brasil

**Keywords:** artroplastia de quadril, complicações pós-operatórias, instabilidade articular, luxações articulares/prevenção & controle, arthroplasty, replacement, hip, joint dislocations/prevention & control, joint instability, postoperative complications

## Abstract

A instabilidade é uma complicação desafiadora e uma das principais causas de revisão na cirurgia de prótese de quadril. A incidência da luxação varia de 0,5 a 10% nas próteses primárias e pode chegar a 30% nas cirurgias de revisão. Existem diversos fatores de risco descritos na literatura, sendo que podemos dividi-los em dependentes do cirurgião, dependentes do paciente e dependentes do implante. O conhecimento pleno desses fatores é fundamental para a prevenção e manejo da instabilidade. O tratamento preventivo envolve planejamento pré-operatório, posicionamento adequado dos componentes, restabelecimento da biomecânica normal do quadril, identificação de fatores de risco e escolha adequada do implante. Aproximadamente dois terços dos episódios de luxação podem ser tratados não cirurgicamente com redução fechada, orientações e fortalecimento muscular. Um terço evoluiu para luxações recorrentes e necessita de intervenção cirúrgica. A cirurgia de revisão deve ser direcionada diretamente para a causa da instabilidade. Quando necessário, considerar a utilização de implantes especiais como acetábulos de dupla mobilidade, insertos de polietileno com rebordo elevado, acetábulos constritos ou cabeças de grande diâmetro. Avanços tecnológicos na área da cirurgia robótica e na compreensão da biomecânica da luxação relacionada ao balanço espinopélvico são temas atuais promissores que devem impactar em melhorias na prevenção e no tratamento da instabilidade.

## Abstract (en)

Instability is a challenging complication and a significant revision cause in hip replacement surgery. The incidence of dislocation ranges from 0.5 to 10% in primary prostheses and can reach up to 30% in revision surgeries. The literature describes several risk factors, including surgeon-, patient-, and implant-related factors. Knowledge of these factors is essential to preventing and managing instability cases. Preventive treatment involves preoperative planning, adequate component positioning, normal hip biomechanics re-establishment, risk factor identification, and proper implant selection. Approximately two thirds of dislocation episodes are treatable with non-surgical treatment with closed reduction, education, and muscle strengthening. One third of the cases develop recurrent dislocations and require surgical intervention. Revision surgery should target the cause of instability. When necessary, consider special implants, such as dual-mobility acetabular components, polyethylene-based lipped acetabular liners, constrained acetabular inserts, or large-diameter prosthetic heads. Technological advances in robotic surgery and the understanding of the biomechanics of spinopelvic swing-related dislocation are promising current topics that may improve the prevention and treatment of instability.

## Introdução

A instabilidade na artroplastia total do quadril é uma complicação significativa e desgastante, sendo uma das principais causas de revisão cirúrgica na atualidade. 1 Embora não exista um registro nacional de dados no Brasil, informações provenientes de países que possuem tais bancos de dados mostram que a instabilidade é uma causa importante de revisão. Por exemplo, o registro australiano classifica a instabilidade como a terceira causa de revisão, representando 14,6% do total; 2 o registro dinamarquês a considera a segunda causa, com uma taxa de 17%; 3 e nos Estados Unidos, a instabilidade é apontada como a principal indicação para revisão, com 17,3%. 4

A incidência de luxação após a artroplastia primária do quadril varia consideravelmente na literatura, dependendo da população estudada, com taxas que oscilam entre 0,5 e 10%. 5 Em casos de artroplastia de revisão, essa incidência pode chegar a até 30%. 6 Historicamente, nas primeiras séries de casos de Charnley, a incidência de luxação reportada era de 4,8%; 7 no entanto, esse número vem diminuindo ao longo do tempo. Uma metanálise de van Erp et al., 8 que examinou a incidência de luxação de 1960 a 2020, indica que a média de luxação pós-artroplastia total do quadril era de 3,7% entre 1960 e 1970, e diminuiu para 0,7% entre 2010 e 2020. Essa redução provavelmente reflete os avanços técnicos e conceituais na abordagem e prevenção da instabilidade.

Na maioria das séries de casos, observa-se que entre 60 e 70% das luxações ocorrem nas primeiras 6 semanas após a cirurgia. 9 Após esse período, o risco cumulativo de luxação é estimado em 1% a cada 5 anos a partir da cirurgia inicial. 7 As luxações tardias podem ocorrer devido a fatores predisponentes que se manifestam tardiamente, como o posicionamento inadequado dos componentes, ou a novos fatores de risco, como o desgaste do polietileno e o desenvolvimento de condições neurológicas. 7

## Fatores de Risco

Diversos fatores de risco para instabilidade estão descritos na literatura, alguns com suporte estatístico mais robusto, enquanto outros apresentam certo grau de questionamento quanto à sua real associação com os episódios de luxação. De maneira prática, os fatores de risco para instabilidade podem ser classificados em três categorias: fatores relacionados ao paciente, ao cirurgião e aos implantes.

### Fatores Relacionados ao Paciente

#### Sexo

O sexo feminino sempre foi associado a um maior risco de instabilidade; 10 porém, revisões sistemáticas mais recentes e dados de registros nacionais têm evidenciando que não há associação direta de episódios de luxação com o sexo do paciente. 1 Provavelmente, vieses de confusão relacionados à maior associação do sexo feminino com diagnósticos como displasia do desenvolvimento do quadril, fraturas do colo femoral e tamanhos de acetábulos menores – que impedem a utilização de cabeças com diâmetros maiores – podem justificar essas discrepância entre os artigos na literatura.

#### Idade

Diversos estudos relatam um maior risco de luxação em pacientes de idade mais avançada, embora não haja consenso sobre a faixa etária específica que está associada a esse risco. 11 Esse fenômeno pode ser compreendido pela presença de várias condições relacionadas à fisiologia do envelhecimento, como a perda de mobilidade da coluna lombossacra, sarcopenia, maior risco de quedas e o desenvolvimento de comorbidades que afetam o sistema neurológico. Curiosamente, alguns estudos recentes indicam que, em faixas etárias mais jovens, especialmente antes dos 50 anos, também pode haver um aumento do risco de luxação. 12 Esse fenômeno pode ser justificado por uma maior demanda funcional nessa faixa etária, além da possível presença de patologias articulares mais complexas nessa população.

#### Doenças Neuromusculares e Insuficiência da Musculatura Abdutora

Este grupo de pacientes engloba diversas patologias que por diferentes mecanismos podem aumentar a probabilidade de luxação por insuficiência da musculatura abdutora, contraturas musculares, perda de mobilidade da coluna lombossacra e falta de cooperação com movimentos de risco para luxação. Esse complexo grupo é representado em sua maioria por pacientes com doença de Parkinson, paralisia cerebral, esclerose múltipla, sequela de poliomielite, sequela de acidente vascular cerebral e lesados medulares, sendo que o risco de luxação nessas situações pode chegar, em algumas séries, de 7,5 até 10,6% dos casos. 13 A insuficiência da musculatura abdutora pode ocorrer em outras situações que não relacionadas a doenças neuromusculares mas que também podem agregar um risco de instabilidade como em casos de revisão com pseudoartrose ou osteólise maciça do trocanter maior, desartrodeses e desinserções tendíneas do glúteo médio extensas. 14

#### Obesidade

Definido como índice de massa corpórea (IMC) acima de 30 kg/m ^2^ , a obesidade sempre foi associada com aumento no risco de luxação podendo apresentar uma razão de risco 2,5 vezes em relação à população normal. 15 Todavia, alguns trabalhos mais recentes colocaram em dúvida essa questão uma vez que não encontraram associação em relação ao índice de massa corpórea e os episódios de luxação. 5 11 Um possível motivo para um risco maior de luxação em pacientes obesos poderia ser o posicionamento inadequado dos componentes decorrente de dificuldades técnicas relacionadas à via cirúrgica pelo tecido subcutâneo mais volumoso; porém, até o momento não conseguimos realizar essa correlação de forma estatisticamente significativa. 1 Curiosamente, uma coorte retrospectiva de mais de 150 mil pacientes apresentou dados que sugerem maior risco de luxação em pacientes com IMC menor que 20 kg/m ^2^ . 10 Raramente trabalhos avaliam de forma separada pacientes com IMC baixos na avaliação da luxação e talvez seja um fator subestimado na literatura.

#### Balanço Espinopélvico

Há pouco mais de uma década, o conceito do balanço espinopélvico, que compreende a relação da mobilidade da coluna lombossacra com o posicionamento espacial do acetábulo, surgiu de forma crescente como mais um fator determinante na estabilidade pós artroplastia do quadril. 16 A mudança de posicionamento da versão pélvica quando estamos sentados ou em pé protege a prótese de se deslocar e, em pacientes que apresentam rigidez desse sistema, como pós operatórios de artrodese da coluna lombossacra ou anquiloses como na espondilite anquilosante, o risco pode aumentar consideravelmente. 17 Um trabalho interessante de Buckland et al., 18 que compara pacientes submetidos à artroplastia total do quadril sem patologias da coluna lombar a aqueles que se submeteram à artrodese, analisou a quantidade de níveis artrodesados e demonstrou que quanto maior o número de níveis, maior o risco de luxação. Esse risco relativo pode chegar a 2,7 vezes na presença de 3 ou mais níveis fixados. Embora ainda não se saiba ao certo como e em que medida essa rigidez do balanço espinopélvico deve influenciar o posicionamento habitual da prótese total do quadril, 19 esse tema representa um campo promissor para avanços científicos futuros.

#### Patologias Específicas

Alguns diagnósticos estão associados a maior risco de luxação, como osteonecrose da cabeça femoral e doenças reumatológicas. 15 Segundo Yang et al., 20 o risco de ocorrer um episódio de luxação após 1 ano de cirurgia de artroplastia total de quadril realizada em caso de osteonecrose é 1,48 vezes maior em relação a pacientes com diagnóstico de artrose primária. Esse dado está de acordo com outra revisão sistemática com metanálise mais recente que encontrou um risco relativo 1,5 maior para casos de osteonecrose. 21 A displasia do desenvolvimento do quadril é um assunto complexo uma vez que apresenta uma variabilidade de apresentações clínicas, dificuldades técnicas e abordagens diferentes para reconstrução óssea; porém, as evidências mais recentes não demonstraram associação com maior risco de instabilidade. 22 Todavia, segundo Komiyama et al. 23 o risco de luxação é maior quando a reconstrução acetabular é realizada 2,39 cm ou mais acima do seu centro fisiológico.

Outro fator de risco importante são as artroplastias realizadas devido a fraturas do colo femoral, que podem apresentar um risco até 10 vezes maior de luxação quando comparado a cirurgias eletivas. 11 13 O perfil do paciente que sofre fratura do colo femoral está normalmente relacionado ao conceito de idoso frágil que acaba por reunir diversos fatores de risco mencionados previamente, como idade avançada, alterações neurológicas/cognitivas, fraqueza da musculatura abdutora e rigidez da coluna lombossacra. Nesse contexto é comum o cirurgião optar por utilizar implantes que tenham menor risco de luxação como próteses constritas, de dupla mobilidade ou até mesmo as hemiartroplastias.

### Fatores Relacionados ao Cirurgião

#### Via Cirúrgica

Historicamente, a via posterolateral sempre foi associada com um risco aumentado de luxação, provavelmente devido ao fato de ser a única via clássica que expõe a articulação através da abertura da cápsula posterior. Entretanto, trabalhos mais recentes que avaliam o risco de luxação da via posterior com a realização do reparo capsular apresentam incidência similar de instabilidade. 24 Segundo a revisão sistemática realizada por Kwon et al., 25 o risco relativo de luxação no acesso posterolateral sem reparo poderia chegar a ser 8,2 vezes maior dependendo da série de caso avaliada; porém, ao comparar a abordagem posterolateral com reparo à abordagem anterior direta e à abordagem lateral, não se observa diferença estatística significativa. Essa conclusão é corroborada por metanálises mais recentes que avaliam as diferentes abordagens cirúrgicas. 26 Existe uma tendência atual à implementação e ao desenvolvimento de vias que tentam poupar mais os músculos e tendões, como a via anterior direta e, mais recentemente, as abordagens Supercapsular Percutaneously-Assisted Total Hip (SuperPATH) e a Save the Piriformis And obturator Internus, Repair Externus (SPAIRE), 27 mas não existe evidência relevante que demonstre superioridade de estabilidade nesses acessos. 28

#### Posicionamento dos Componentes

Classicamente a zona de segurança descrita por Lewinnek é o parâmetro mais amplamente difundido quando se estuda o posicionamento dos componentes sugerindo uma inclinação acetabular de 40 ± 10° e anteversão de 15 ± 10°. 29 Entretanto, existem muitas críticas a respeito desses parâmetros, uma vez que o artigo apresenta diversos vieses e também devido a muitos trabalhos posteriores não conseguirem demonstrar uma reprodutibilidade estatística dos achados de Lewinnek. 30 Outro conceito famoso é o da anteversão combinada apresentada através do teste de Ranawat descrito em 1994, 31 que consiste na soma das versões do fêmur e do acetábulo, sendo que Dorr et al. 32 definiram, através de um amplo estudo utilizando navegação, que a zona de segurança da anteversão combinada estaria entre 25 e 50°. Mesmo respeitando todos esses parâmetros, as luxações ainda podem ocorrer, tanto que alguns autores afirmam que não se pode definir uma zona de segurança para o posicionamento dos componentes. 5 A instabilidade é multifatorial e muitas vezes é complexo conseguir isolar de forma efetiva os diversos fatores de risco que podem levar à luxação, incluindo variações anatômicas individuais. Talvez com avanços tecnológicos relacionados a tomografias em três dimensões e análises biomecânicas que levem em consideração o balanço sagital e variações individuais poderemos definir o posicionamento ideal de forma personalizada para cada paciente no futuro.

#### Restabelecimento da Anatomia

A reconstrução dos parâmetros anatômicos fisiológicos de *offset* , comprimento e centro de rotação do quadril é imperativa para um bom funcionamento biomecânico da prótese e, consequentemente, evitar os impactos entre os componentes protéticos e estruturas ósseas do paciente que levam à luxação. Para isso, é fundamental que seja realizado um planejamento cirúrgico minucioso com a escolha de implantes que permitam o restabelecimento da anatomia fisiológica do paciente. Atualmente estamos vivenciando uma transição entre o planejamento em radiografias impressas e o planejamento digital. Existem diversas dificuldades neste processo adaptativo, mas com o tempo a tecnologia está se aperfeiçoando e cada vez mais o planejamento digital será mais preciso. 33

### Fatores Relacionados ao Implante

#### Tamanho da Cabeça Femoral

O tamanho da cabeça femoral é um fator bastante abordado na literatura, uma vez que o diâmetro da cabeça influência na amplitude de movimento e na *jumping distance* . 34 A maioria dos estudos demonstra que cabeças femorais menores ou iguais a 28 mm são fatores de risco para luxação, enquanto que cabeças iguais ou maiores a 32 mm são fatores de proteção. 35 Todavia existem questionamentos se haveria real benefício em utilizar cabeças maiores do que 36 mm devido a questões relacionadas ao desgaste volumétrico do polietileno, risco de fratura do inserto acetabular, impacto da cabeça em partes moles que pode causar dor inguinal, além de preocupações como a truniose e reação tecidual adversa local.

#### Insertos Acetabulares com Rebordo Elevado

A utilização de insertos de polietileno com rebordo elevado pode ser um fator de proteção em casos de maior risco de luxação, aumentando a distância que a cabeça femoral necessita percorrer para desacoplar do componente acetabular. 6 Apesar de haver alguma preocupação com a possibilidade de impacto do componente femoral contra a o rebordo elevado do polietileno que poderia causar um aumento paradoxal da luxação e risco de soltura precoce, revisões sistemáticas e estudos de coorte não evidenciaram complicações associadas a estes implantes. 36

#### Tribologia

O desgaste do componente acetabular de polietileno está associado com episódios de luxação tardia, principalmente após 5 anos da cirurgia inicial, 7 uma vez que a frouxidão ligamentar ocasionada pela ascensão da cabeça no rebordo desgastado e a microinstabilidade local poderia levar à luxação. 13 Dessa forma foi sugerido por alguns autores a utilização do par tribológico de cerâmica/cerâmica como alternativa para evitar a luxação tardia. Entretanto, com o avanço do polietileno reticulado, o desgaste a longo prazo se tornou menor e não influencia mais o risco cumulativo, como é possível observar em registros de dados nacionais. 37

#### Acetábulos Constritos

Os acetábulos constritos englobam uma variedade de implantes que compartilham um mecanismo de travamento da cabeça femoral no inserto de polietileno. A literatura apresenta uma controvérsia considerável quanto ao seu uso: algumas séries relatam excelentes resultados, com taxas de falha entre 1,8 e 2,4% em um tempo de seguimento médio de 4 a 5 anos, especialmente em pacientes de alto risco de instabilidade. 38 39 Por outro lado, outras investigações sugerem que esses implantes devem ser utilizados apenas como procedimentos de salvamento, apresentando taxas de falha que variam de 10 a 23%. 40 41

A principal preocupação relaciona-se à limitação da amplitude de movimento dentro de valores fisiológicos, que pode gerar estresse excessivo na interface implante/osso ou entre o componente femoral e o mecanismo de travamento. Isso pode resultar em soltura precoce, desgaste acelerado do polietileno ou falha do mecanismo de constrição com consequente falha do implante. 40 É importante ressaltar que existem diversos designs de implantes com diferentes mecanismos de travamento, e cada um deles terá desfechos distintos. 42 Implantes que restringem menos a amplitude de movimento geralmente apresentam resultados mais positivos, como é o caso dos implantes tripolares, 43 bem como dos implantes que possuem reentrâncias nas áreas mais comuns de impacto, que visam reduzir a restrição do movimento. 44

#### Acetábulos de Dupla Mobilidade

O conceito da prótese de dupla mobilidade não é recente, sendo desenvolvido por Bousquet et al. 45 em 1973 na França. Todavia, decorrente de complicações relacionadas ao desgaste precoce do polietileno e da alta incidência de luxação intraprotética dos primeiros dispositivos, 46 a literatura sobre a dupla mobilidade só veio a ganhar destaque de forma difusa a partir de 2009, quando foi liberada pela Food and Drug Administration (FDA) nos Estados Unidos. 47 Com avanças tecnológicos no design dos implantes (atualmente estamos na terceira geração) e com o advento do polietileno reticulado, essas complicações diminuíram de forma substancial sendo que a metanálise mais recente que avalia próteses primárias eletivas demonstra uma sobrevida para qualquer causa de revisão de 99,7% em 5 anos e 96,1% em 15 anos. 48 Quando comparado com os acetábulos constritos, há uma tendência na literatura de favorecer a dupla mobilidade como é demonstrado por outra revisão sistemática com metanálise de Donovan et al., 49 que evidencia uma falha para luxação de 3% para a dupla mobilidade versus a 9% para constrita, uma taxa de revisão por instabilidade de 2% versus 9% e uma taxa de revisão por qualquer causa de 8% versus 19%. Contudo, ainda existe uma preocupação com a sobrevida a longo prazo e falha em casos especiais como na insuficiência grave do mecanismo abdutor. 50

#### Navegação e Robótica

A navegação cirúrgica na artroplastia total do quadril foi introduzida em 1998 por Jaramaz et al. 51 e consiste em um sistema no qual a posição espacial do paciente (baseado em referências anatômicas fornecidas pelo cirurgião no intraoperatório) é digitalmente capturada e processada por um software especializado que fornece ao cirurgião informações visuais, gráficas ou numéricas em tempo real sobre as etapas do procedimento, ampliando o controle e a precisão do posicionamento dos componentes. 52 Apesar dos trabalhos se mostrarem promissores em relação à precisão do posicionamento acetabular, a literatura falhou em demonstrar evidência de melhora no desfecho clínico desses pacientes, 53 fazendo com que a técnica não se difundisse. Mais recentemente, a robótica associou o conceito de navegação à utilização de braços robóticos que assistem o cirurgião no posicionamento dos componentes e no restabelecimento da anatomia prévia. Apesar dos trabalhos evidenciarem maior precisão em posicionar os componentes na zona de segurança na cirurgia robótica assistida (97%) quando comparada com a navegação (84%) e métodos manuais (74%), 54 questões a respeito do custo benefício dessa técnica, levando em consideração o valor do robô e o custo operacional, colocam em dúvida a vantagem de se adotar de forma sistemática essa técnica. 55 Todavia, trabalhos recentes estão cada vez mais demonstrando diminuição na incidência de luxação com o auxílio de cirurgia robótica, principalmente em casos complexos com disfunção do balanço espinopélvico. 56 O futuro é bastante promissor a respeito de técnicas robóticas, principalmente no cenário atual do desenvolvimento de tecnologias de inteligência artificial.

## Prevenção

O tratamento preventivo da instabilidade requer a otimização técnica de todos os fatores de risco modificáveis, ou seja, que dependem do cirurgião. Os elementos a serem considerados incluem:

- Planejamento pré-operatório minucioso com o intuito de reconstruir de forma precisa o posicionamento fisiológico do centro de rotação acetabular, *offset* global e equalização de membros.
- Acesso cirúrgico que respeite a integridade dos tecidos (independente da via de escolha), o que demanda um cuidado especial na manipulação e reinserção tendínea, muscular e um adequado reparo capsular.
- Posicionamento adequado dos componentes dentro das zonas de segurança e anteversão combinada.
- Identificação de todos os fatores de risco inerentes ao paciente, a fim de escolher mais adequadamente os implantes que podem minimizar os riscos de luxação. Apesar desses fatores não serem modificáveis, podemos optar por implantes com cabeças de grande diâmetro, acetábulos com elevação do rebordo, dupla mobilidade ou constritos. Cada um apresenta vantagens e desvantagens que devem ser levadas em consideração individualmente.

## Tratamento Não Cirúrgico

Aproximadamente dois terços das luxações podem ser tratados de forma não cirúrgica com bons resultados, principalmente nos casos de luxações que ocorrem nos primeiros 3 meses e nas quais o posicionamento dos componentes e o restabelecimento anatômico estão adequados. 13 Uma vez obtida a redução fechada, medidas de orientação do paciente a respeito de movimentos de risco para instabilidade e fisioterapia motora com objetivo de fortalecimento muscular dos estabilizadores do quadril são fundamentais. Em situações especiais de falta de cooperação do paciente ou insuficiência muscular relevante, pode-se utilizar órteses de abdução do quadril por 8 a 12 semanas, mas a literatura demonstra falta de evidências que favoreçam o uso rotineiro desses dispositivos. 57

## Tratamento Cirúrgico

A revisão da artroplastia está indicada em casos de luxação recorrente. Apesar de não existir um número definido de episódios de luxação que indique de forma absoluta uma intervenção cirúrgica, luxações tardias ou casos em que existe mal posicionamento dos componentes estão mais associados à recorrência e devem ter um limiar menor para indicação da revisão.

A conduta cirúrgica nesses casos está diretamente relacionada com a causa da instabilidade. Dessa forma, é fundamental uma análise criteriosa de todos os fatores de riscos apresentados pelo paciente, entendendo que muitas vezes a causa é multifatorial. Para nos auxiliar na tomada de decisão de forma prática, podemos utilizar a classificação descrita por Wera et al., 58 que classifica as instabilidades em 6 tipos ( Tabela 1 ) e as orientações de manejo dessas instabilidades apresentadas por Sheth et al. 59

**Tabela 1:** Classificação etiológica da instabilidade 58

Tipo I	Posicionamento inadequado do componente acetabular
Tipo II	Posicionamento inadequado do componente femoral
Tipo III	Insuficiência da musculatura abdutora
Tipo IV	Impacto ósseo ou partes moles
Tipo V	Desgaste assimétrico do polietileno
Tipo VI	Sem causa identificável

Nas instabilidades tipo I (mal posicionamento do componente acetabular) e II (mal posicionamento do componente femoral), está indicada a revisão do componente em questão com otimização do restabelecimento dos parâmetros anatômicos. A avaliação do posicionamento dos componentes pode ser feita através de radiografias convencionais com incidências variadas; porém, a utilização da tomografia computadorizada auxilia de forma mais precisa a entender esses erros de posicionamento. 53

Na instabilidade do tipo III, ne qual existe uma insuficiência da musculatura abdutora, a revisão com uso de acetábulos constritos está indicada. 59 A utilização de acetábulos de dupla mobilidade nesse cenário de insuficiência importante do mecanismo abdutor tem se mostrado inconsistente. 50 Se possível deve-se minimizar a insuficiência do complexo abdutor realizando, por exemplo, reparando desinserções tendíneas dos glúteos médio e mínimo e estabilizando pseudoartroses do trocanter maior. Técnicas que envolvem transferências musculares, reconstruções com aloenxerto e osteotomias trocantéricas de distalização são descritas na literatura relacionadas à melhora nos padrões funcionais, mas sem clareza se apresentam algum impacto no tratamento da instabilidade. 14

Na instabilidade tipo IV a luxação ocorre por algum impacto que limita a amplitude do arco de movimento, podendo ser ósseo ou relacionado a contratura de partes moles periarticulares que não estão bem balanceadas. 59 É fundamental a identificação da causa do impacto e sua remoção. A utilização de implantes que aumentem a amplitude de movimento, como cabeças de grandes diâmetros ou acetábulos de dupla mobilidade, é desejável nesses casos.

Na instabilidade tipo V, a causa é um desgaste assimétrico do inserto de polietileno, que deve ser revisado. Se possível, a utilização de cabeças grandes com polietileno altamente reticulado é desejável para minimizar a recorrência da instabilidade. Em componentes fixos nos quais não existe a possibilidade de troca modular do inserto de polietileno por questões relacionadas à disponibilidade do implante, é possível realizar a cimentação dentro de uma cúpula de metal fixa com resultados promissores. 60

Na instabilidade tipo VI, não existe causa aparente modificável para justificar os episódios de luxação e o tratamento sugerido é a revisão com utilização de acetábulos constritos.

## Considerações Gerais

A instabilidade na artroplastia total do quadril é uma das principais complicações na atualidade, sendo sua etiologia complexa e multifatorial. A compreensão dos fatores de risco, o conhecimento a respeito dos diversos implantes disponíveis e um planejamento preciso e individualizado é fundamental para a prevenção e tratamento dessa desafiadora complicação.
